# The Use of *Hanseniaspora opuntiae* to Improve ‘Sideritis’ Wine Quality, a Late-Ripening Greek Grape Variety

**DOI:** 10.3390/foods13071061

**Published:** 2024-03-29

**Authors:** Maria-Evangelia Filippousi, Ioanna Chalvantzi, Athanasios Mallouchos, Ioannis Marmaras, Georgios Banilas, Aspasia Nisiotou

**Affiliations:** 1 Institute of Technology of Agricultural Products, Hellenic Agricultural Organization “Dimitra”, Sofokli Venizelou 1, 14123 Lykovryssi, Greece; mariliafilippousi@gmail.com (M.-E.F.); ichalvantzi@uniwa.gr (I.C.); ioannismarmaras.ga@gmail.com (I.M.); 2 Department of Wine, Vine and Beverage Sciences, University of West Attica, Spyridonos 28, 12243 Athens, Greece; gban@uniwa.gr; 3 Laboratory of Food Chemistry and Analysis, Department of Food Science and Human Nutrition, Agricultural University of Athens, Iera Odos 75, 11855 Athens, Greece; amallouchos@aua.gr

**Keywords:** non-*Saccharomyces*, wine fermentation, yeast starter cultures, *Hanseniaspora opuntiae*, wine chemical profile

## Abstract

In view of climate change and the increasingly antagonistic wine market, the exploitation of native genetic resources is revisited in relation to sustainable wine production. ‘Sideritis’ is a late-ripening Greek grape variety, which is quite promising for counteracting wine quality issues associated with the annual temperature rise. The aim of this study was to improve the quality and to enhance the aroma of ‘Sideritis’ wine through the use of native yeasts. To improve vinification, *Hanseniaspora opuntiae* L1 was used along with *Saccharomyces cerevisiae* W7 in mixed fermentations (SQ). The addition of *H. οpuntiae* significantly altered the chemical profile of the wine compared to the single-inoculated fermentations with W7 (IS). *H. opuntiae* increased all the acetate esters, except for hexyl acetate and (Z)-3-hexen-1-ol acetate. The concentration of 2-phenylethyl acetate, which imparts flowery and sweet notes, exhibited a 2.6-fold increase in SQ as compared to IS wines. SQ also showed higher levels in several ethyl esters, including ethyl butyrate, ethyl heptanoate and ethyl 7-octenoate, which are associated with fruity notes compared to IS. *H. opuntiae* produced citronellol, a terpene associated with rose and green notes, and increased the overall acceptance of the wine. Present results are thus quite promising for improving ‘Sideritis’ wine quality towards a sustainable wine production in Greece in view of global warming.

## 1. Introduction

Wine production is currently facing emerging challenges such as climate change and an increasingly antagonistic wine market. Climate change is evident by an increase in severe weather phenomena and the elevation of the average yearly temperature to which grapevine is quite vulnerable. In response to climatic changes, the composition of the grape juice may be altered leading to a diminishment in wine quality. Global warming, in particular, is anticipated to decrease the acidity while boosting the sugar levels in berries, resulting in unbalanced wines with elevated alcohol content that are deprived of fruitiness and aromatic complexity [1]. In the long run, the sustainability of winegrowing and the suitability of international grape cultivars in traditional viticultural regions of warm climates will become questionable. It is foreseen that Mediterranean winegrowing regions will be particularly susceptible to these changes, leading to higher-altitude vineyard sites, northward migration or introduction of grape varieties better suited to warmer climates [1,2].

The exploitation of native germplasm adapted to stress conditions related to climate change, such as global warming, is currently revisited as an alternative approach to sustainable wine production. ‘Sideritis’ is a rare Greek grape variety (*Vitis vinifera*) that has extremely late ripening and is well-adapted to global warming [1] and is lately claiming a place in modern viticulture [3]. It is a vigorous and quite productive variety, bearing large branches of pink-coloured hardy berries. It produces cool light white or rose wines with a “crunchy” acidity. Nevertheless, it is seldom used for the production of varietal wines due to the relatively low aroma intensity and diminished complexity of the produced wines. Therefore, means for the improvement of its aroma and the overall quality would be of great interest for the sustainability of the local wine industry in view of global warming.

The exploitation of native microbial genetic resources may also provide a powerful means to address these novel challenges in winemaking. In this context, the use of novel *Saccharomyces cerevisiae* strains and non-*Saccharomyces* (NS) yeasts is being considered towards wine improvement. Although NS yeasts have been previously considered unwanted in winemaking, due to the production of off-odours and flavours, certain NS strains have been shown to enhance the complexity and the organoleptic profile of wines [4]. As more data becomes available, it appears that various NS yeasts exhibit significant technological characteristics which are to be considered for the development of new starter cultures adapted to the new challenges [5]. Examples of the beneficial activities of NS yeasts include the reduction of volatile acidity or ethanol content, the enhancement of acidity, the production of various aroma compounds, and colour stabilization [6,7]. However, most relevant studies have been conducted at a laboratory scale, including sterile or synthetic musts and small volumes of fermentations, that makes it difficult to conduct reliable sensory analysis and judge the final product in terms of real market wine.

Among the several NS yeasts encountered in grape must, *Hanseniaspora opuntiae* is a relatively recently described apiculate species [8]. It is quite common in Greek vineyards as part of the NS wine yeast flora [9] where it was first found to be associated with grapes and the initial stages of alcoholic fermentations [10]. Different *Hanseniaspora* spp., mostly *H. osmophila* and *H. vineae*, but also *H. guilliermondii* and *H. uvarum*, have been evaluated as potential partners of *S. cerevisiae* in winemaking [11,12]. However, there is scarce information on the technological characterization of *H. opuntiae*, while its potentially positive role in winemaking has not been thoroughly evaluated as yet. Certain *H. opuntiae* strains have been shown to decrease the ethanol content of wines, while increasing the glycerol level [13] or to increase higher alcohols (phenylethanol and 3-methylbutanol) and phenylacetaldehyde in co-culture with *S. cerevisiae* [14]. Recently, the aroma production profiles of seven different strains of respective *Hanseniaspora* species, including *H. opuntiae*, was assessed in simultaneous co-inoculation with *S. cerevisiae* in microvinifications of Gewürztraminer grape must [15]. *H. opuntiae* was found to increase certain terpenes, such as citronellol and β-myrcene. Yet, limited information on the influence of *H. opuntiae* metabolism on the resulting wine, also considering that its impact may vary among different strains, grape cultivars, geographical regions, and winemaking practices.

Here we evaluated the performance of two native yeasts, *H. opuntiae* and *S. cerevisiae*, to enhance the aroma profile of wine produced by the late ripening ‘Sideritis’ grape variety. Alcoholic fermentations were conducted outside laboratory conditions, like those encountered in a winery. Therefore, the present results are quite promising for winemaking alternatives in the era of climate change, towards the production of a new brand on the market made by solely ‘Sideritis’ grapes.

## 2. Materials and Methods

### 2.1. Alcoholic Fermentations

Yeast strains *Hanseniaspora οpuntiae* L1 (hereafter referred to as L1) and *Saccharomyces cerevisiae* W7 (hereafter referred to as W7) from our culture collection at the Institute of Technology of Agricultural Products and the commercial starter *S. cerevisiae* VIN13 marketed by “Oenobrands SAS, France” were used in the present study. The strains were previously isolated from spontaneously fermented grape musts from Santorini island (L1) and Achaia region in Peloponnese (W7). Fermentations were carried out in duplicate in 20-L vessels at 16 °C with unfiltered ‘Sideritis’ grape must (*Vitis vinifera*) originating from the Achaia region of western Peloponnese, Greece, with the following characteristics: sugars 176 g/L, pH 3.27, yeast assimilable nitrogen 246 mg/L, total SO_2_ 30 ppm as potassium metabisulfite (K_2_S_2_O_5_). Yeasts were grown in yeast extract peptone dextrose (YPD) agar at 26 °C and then resuspended in 1/4 strength Ringer’s solution. The following inoculation protocols were applied: single-culture inoculation with the indigenous strain *S. cerevisiae* W7 (IS) or with the commercial *S. cerevisiae* VIN13 (SC), and sequential inoculation (SQ) with strain L1 followed by strain W7 after ca. 1% vol ethanol production (at 8 h). The size of inoculum was at 6 log CFU/mL at single-culture inoculation. In mixed-culture fermentations, *S. cerevisiae* and *H. opuntiae* were added at 5 log CFU/mL and 7 log CFU/mL, respectively. Spontaneous (un-inoculated) fermentations (SP) were performed as a reference. Fermentation progress was monitored by density measurements. Seventy ppm of the total SO_2_ was added to the finished wines at the end of the alcoholic fermentations. Wines were stored at 4 °C in fully filled containers for one week before being bottled and chemically analysed.

### 2.2. Microbiological Analysis

Grape must samples were taken at different time intervals during fermentation for estimating yeast populations. One ml of samples was serially diluted and plated on Wallerstein laboratory nutrient agar (WL), ethanol sulfite agar (ESA) or lysine medium agar (LA) for the enumeration of total yeasts, *S. cerevisiae* and non-*Saccharomyces* species, respectively. Plated samples were incubated at 28 °C for 2–5 days. Yeast colonies were isolated from the initial, middle, and final stages of fermentations and examined microscopically. For *S. cerevisiae* genotyping, the interdelta region analysis using the primer set delta 12/delta 21 was applied [16]. For genotyping NS yeasts, the tandem repeat-tRNA method using the primer pair TtRNASc/ISSR-MB was applied [17].

### 2.3. Chemical Analysis

Reducing sugars, total acidity, volatile acidity, pH, ethanol, total SO_2_ and free SO_2_ were estimated using the methods in the Compendium of International Methods of Analysis of Musts and Wines [18]. Yeast assimilable nitrogen (YAN) was determined in the grape must as described previously [19]. Malic acid, lactic acid, citric acid, glycerol, and acetaldehyde were measured in a wine automatic analyser (Miura One, TDI, Barcelona, Spain). The volatile compounds were extracted by headspace solid-phase microextraction (SPME) and analyzed by gas chromatography–mass spectrometry (GC–MS) as described [20]. Compounds were identified by comparing the following: (i) retention indices (RI) based on the homologous series of C8–C24 n-alkanes with those of authentic compounds (when available) and those of the NIST 14 library (Scientific Instrument Services, Ringoes, NJ, USA); (ii) mass spectrometry (MS) data with those of reference compounds and MS data obtained from NIST14. The concentrations of volatile compounds were calculated relative to the internal standard (1,4-dioxane) and expressed as mg/L.

### 2.4. Sensory Analysis

The quantitative descriptive analysis method was applied for the sensory evaluation of wines. Wines were evaluated about four months after bottling by a group of six skilled evaluators: 3 males and 3 females 30–55 years old, either members of the Institute of Technology of Agricultural Products (ITAP) or the Department of Wine, Vine and Beverage Sciences, University of West Attica), which provided consent prior to their participation. Wines were tested in duplicate in two sessions in random order and at a temperature of 12 °C. Description was based on seven aromas (tree fruits, citric fruits, tropical fruits, floral, fermentation aromas, pungent, aroma intensity) and six palate (oxidation, acidity, sweetness, bitterness, body, after-taste) attributes on a scale from 0 (not perceivable) to 10 (high intensity).

### 2.5. Statistical Analysis

Analysis of Variance (ANOVA) and Tukey’s HSD test were used to assess significant differences in the chemical profiles of the wine. Chemical parameters were subjected to principal component analysis (PCA) in order to analyse interactions between samples and variables. To compare inoculation protocols, a permutational multivariate analysis of variance (PERMANOVA) was applied. Pairwise distances were estimated by Jaccard metric based on 4999 permutations. PAST program version 3.11 [21] and PRIMER Version 7 software (https://www.primer-e.com (accessed on 5 March 2024)) were used for statistical analyses.

## 3. Results

### 3.1. Fermentation Kinetics and Yeast Population Dynamics

**Figure 1:**
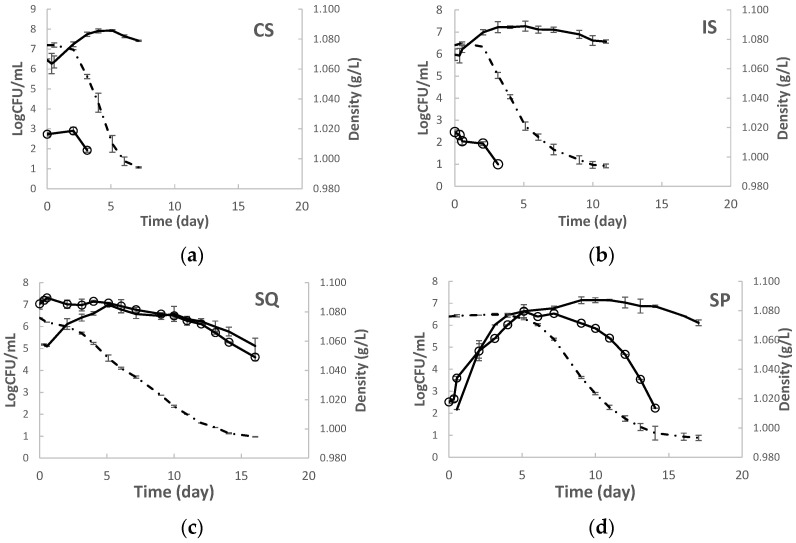
Fermentation kinetics (dashed line) and yeasts population (continuous line) in grape musts inoculated with (**a**) *S. cerevisiae* VIN 13 (CS), (**b**) *S. cerevisiae* W7 (IS), (**c**) *H. opuntiae* L1 and *S. cerevisiae* W7 added sequentially, and (**d**) non-inoculated fermentation (SP). ESA agar was applied for enumeration *S. cerevisiae* (-), and LA (○) for non-*Saccharomyces* yeasts.

Yeast kinetics were followed in a single inoculation of grape must with *S. cerevisiae* W7 (IS) and mixed sequential inoculation (SQ) with *H. opuntiae* L1 followed by W7 (Figure 1). Fermentations were also performed with the commercial *S. cerevisiae* VIN13 (CS) and with non-inoculated must (SP) for comparison reasons. The highest fermentation rate was recorded in CS, followed by the IS fermentation. The SQ fermentation had a markedly lower rate compared to both CS and IS. A significantly prolonged lag phase was observed in SP; yet the rate during the log phase was higher than the one observed in SQ and comparable to both CS and IS. Concomitantly, the SQ and SP fermentations lasted longer (*ca.* 15 d) than IS (*ca.* 12 d), whereas VIN13 was the fastest fermentor (*ca.* 10 d).

The strain W7 showed different kinetics in co-inoculated compared to single-inoculated fermentations (Figure 1). In IS, W7 reached a plateau at 3 d after inoculation at maximum population of 7.27 ± 0.23 Log CFU/mL. In SQ, W7 reached plateau at 5 d after inoculation and exhibited somewhat lower cell density than in IS by 0.5 Log CFU/mL till day 10. The max population of W7 was also lower in SQ than in IS (7.00 ± 0.06 Log CFU/mL). W7 achieved a lower cell density than VIN 13 (7.93 ± 0.04 Log CFU/mL) and similar to the indigenous *S. cerevisiae* counts in SP (7.14 ± 0.15 Log CFU/mL).

Significant differences were observed in the kinetic behaviour of the NS yeast populations in different fermentation trials. In SQ, the strain L1 retained its high population levels till day 6, after which a slow decline was observed. NS yeasts immediately declined upon the addition of *S. cerevisiae* in both the IS and CS fermentations. As opposed to the indigenous NS yeasts which gradually developed in SP, reaching a maximum of 6.64 ± 0.31 Log CFU/mL by date 5. The NS populations started to gradually decline by day 9.

### 3.2. Effect of Different Inoculation Schemes on Wine Chemical Profiles

The chemical characteristics of wines were compared by ANOVA to identify differences between the inoculation protocols applied (Table 1). Significant differences were observed in specific characteristics between the commercial and the native *S. cerevisiae*. The largest differences were observed in volatile acidity (VA) and acetaldehyde levels that were significantly higher in IS than in CS. CS also displayed higher levels of glycerol than IS. The use of *H. οpuntiae* in SQ significantly increased the acetaldehyde levels and lowered the malic acid concentration. The volatile profile of wines was also determined by GC-MS analysis (Table 2). The commercial *S. cerevisiae* (CS) produced higher levels of acetate esters compared to the native strain (IS), except for heptyl acetate and 2-phenylethyl acetate. The most profound differences were observed in propyl acetate, isobutyl acetate and isoamyl acetate (fold change > 1.5). On the other hand, the native *S. cerevisiae* generated more medium-chain fatty acid esters than the commercial strain, such as ethyl heptanoate, ethyl octanoate, ethyl decanoate, 3-methylbutyl octanoate, and ethyl 9-decenoate (fold change > 2). CS and IS wines displayed similar levels of total alcohols. IS produced higher levels (fold change > 2) of 1-decanol, 2-phenylethanol, and 1-heptanol compared to CS, while CS wines had elevated levels of 1-propanol, 2-nonanol, 1-octanol and 2-undecanol (fold change > 1.5). Regarding carboxylic acids, IS wines were characterized by significantly higher contents of acetic, hexanoic and octanoic acid. The addition of *H. οpuntiae* in SQ fermentation significantly altered the volatile profile of the produced wine compared to IS. In more detail, a high rise (fold change ~ 2) in the levels of acetate levels was recorded, mostly due to the production of ethyl acetate and 2-phenylethyl acetate. On the contrary, the IS wines presented a significantly higher total content of fatty acid esters, mostly due to ethyl octanoate, ethyl decanoate and ethyl 9-decenoate. However, the SQ wines presented a higher content (fold change > 1.5) of quite a few minor fatty acid esters compared to IS, such as ethyl propionate, ethyl isobutyrate, ethyl heptanoate and ethyl 7-octenoate. The total content of alcohols and acids was found higher in IS wines than SQ wines. This was due to the increased content of 3-methyl-1-butanol, 2-phenylethanol, octanoic acid and decanoic acid. There were no significant differences in the content of carbonyl compounds that was observed between samples. Citronellol levels in SQ wines were significantly higher (*p* < 0.05) than in IS and CS wines.

**Figure 2:**
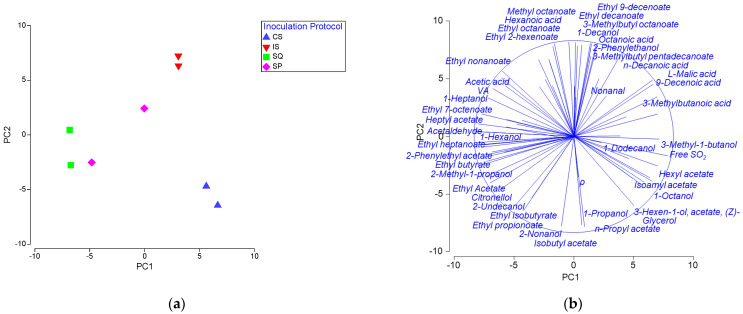
PCA of the chemical profiles of wines: (**a**) PCA score plot; (**b**) PCA loading plot of the chemical characteristics. PC1 and PC2 account for 39.9% and 33.0% of the total variation, respectively. CS: commercial *S. cerevisiae*; IS: *S. cerevisiae* W7; SQ: *H. opuntiae* L1 and *S. cerevisiae* W7 added sequentially; and SP: spontaneous fermentation.

The chemical characteristics and the volatiles of wines were examined by PERMANOVA. It was observed that the inoculation protocol significantly influenced the chemical character of wines (F = 10.2, *p* < 0.05). According to pairwise comparisons, the SQ wine showed the highest similarity to SP (F = 1.4), while it was most dissimilar to CS (F = 35.68). PCA was applied to depict relationships between chemical profiles of wines produced by different inoculation protocols (Figure 2a). The first two principal components represented 72.9% of the overall variability (39.9% and 33.0% for PC1 and PC2, respectively). IS, CS and SQ formed distantly separated clusters on the PCA plot. SP samples were more dispersed to each other compared to other treatments. CS exhibited high values on PC1 for isoamyl acetate, glycerol, and 3-methyl-1 butanol, among other metabolites, which separated it from IS along the PC1 axis and SQ along the PC2 axis. SQ was separated from IS along the PC2 axis due to the high scores for several important metabolites such as ethyl acetate, citronellol and 2-phenylethyl acetate on PC2.

### 3.3. Sensory Analysis

**Figure 3:**
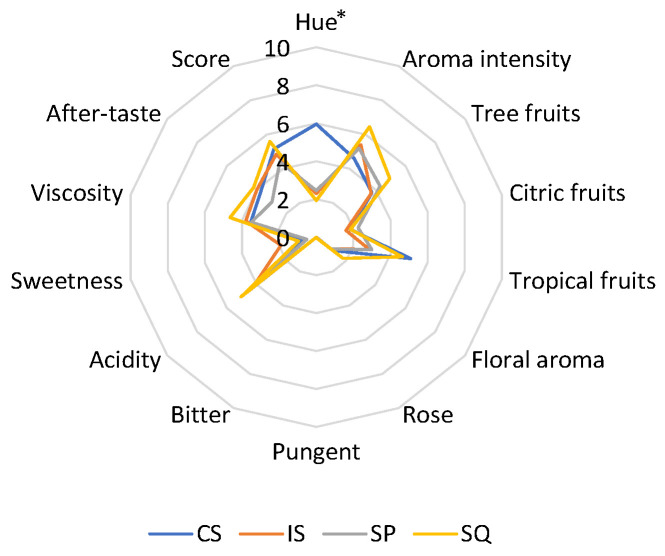
Means of the sensory characteristics of wines. CS: *S. cerevisiae* VIN13; IS: *S. cerevisiae* W7; SQ: *H. opuntiae* L1 and *S. cerevisiae* W7 added sequentially; SP: spontaneous fermentation. Significant differences among samples (*p* < 0.05) are shown by an asterisk.

The average values of the sensory attributes of wines are shown in Figure 3. The SQ wines showed the highest overall aroma intensity and the most notable tree-fruits and floral notes. The use of *H. opuntiae* was also shown to increase the tropical-fruits character in SQ compared to IS. A rose tone was identified in SQ wines, albeit at very low levels. SQ wines also showed increased acidity, viscosity, and after-taste compared to other wines. Pungent character was scored at low levels (average of 0.05) in SQ wines. Overall, the SQ wines received the highest score among all wines tested by the panel of accessors.

## 4. Discussion

The use of indigenous grape and microbial genetic resources is being reconsidered as an alternative strategy for sustainable wine production in light of the impending climate change and the wine market’s growing antagonism. A unique Greek grape variety called ‘Sideritis’, which is endemic to the Achaia area of Peloponnese, has extremely delayed ripening and thus could effectively combat the yearly rise in temperature [1,22]. Although ‘Sideritis’ c.v. may produce delicate wines, it is rarely utilized to make varietal wine due to the low aroma intensity of the final product. The purpose of this study was to investigate the use of *H. opuntiae*, a relatively unexplored NS wine yeast, in mixed fermentation with *S. cerevisiae* with the aim to enhance the aroma complexity of ‘Sideritis’ wine.

The genus *Hanseniaspora* is predominant in fresh grape must, producing enzymes and aroma compounds that enhance wine flavour [23]. *H. opuntiae*, a recently described member of the *Hanseniaspora* genus, has been shown repeatably to prevail over other *Hanseniaspora* species in grapes from Greek vineyards [9] indicating its predominance as a component of the native wine microbiota. *H. opuntiae* in grape must fermentation has been shown to decrease the cell growth rate of *S. cerevisiae* [14]. In the present study, the addition of *H. opuntiae* in high counts delayed both the rate of development and the maximum population reached by *S. cerevisiae*. Other *Hanseniaspora* species may also retard the growth of *S. cerevisiae* [24] even to a greater extent as compared to other NS yeasts [25]. For instance, *H. uvarum* retarded *S. cerevisiae* more than *L. thermotolerans* [25]. In line with that, *H. opuntiae* and *H. uvarum* show a better capacity to grow and persist in grape must compared to *Pichia kudriavzevii* and *Candida flavescens* [13]. Taken together, it seems that *H. opuntiae* is a good competitor against *S. cerevisiae* as is also the case for other *Hanseniaspora* species. Their death is principally dictated by environmental factors [25,26].

The implication of several secondary metabolic activities of non-*Saccharomyces* yeasts in alcoholic fermentations leads to a heightened sensory complexity in wines [27]. The alternate direction of carbon to metabolic products other than ethanol during the alcoholic fermentation may lower the final ethanol content of wines. It was previously shown that two *H. opuntiae* isolates produced less ethanol per gram of sugar consumed compared to *S. cerevisiae* and significantly reduced the ethanol levels in the final wines [13]. However, *H. opuntiae* did not decrease the ethanol content in favour of glycerol in our study coinciding with previous results [14,15] and suggesting that this capacity could be probably strain-dependent.

Acetaldehyde is an important constituent of wines which at low levels confers fruity notes but it can impart undesirable ‘nut-like’ odours above 125 mg/L in table white wines, or a grassy and apple-like off-flavour at even higher concentrations [28]. In the present study, it was produced in higher concentrations in SQ (100 mg/L) compared to IS fermentations, close to the threshold of perception in the wine (100–125 mg/L). *Hanseniaspora* is known to produce relatively high levels of acetaldehyde which are typically significantly higher than those produced by *S. cerevisiae* [11]. In the present study, acetaldehyde-associated defects were not detected in the sensory analysis. Nevertheless, acetaldehyde production should be taken into consideration if *H. opuntiae* is employed in winemaking.

Yeasts release free fatty acids during fermentation that may add to the overall complexity of wine at low levels, but at increased concentrations they tend to enhance rancid notes [29]. In the present study, six fatty acids were found in wines. The presence of *H. opuntiae* in the inoculum decreased the levels of most fatty acids including those of the toxic octanoic and decanoic acids. The reduction of the latter by the use of other strains of *H. opuntiae* has been also reported recently [13,14]. Therefore, the use of *H. opuntiae* in wine fermentation may be considered as a positive contributor towards the decrease in toxicity and unpleasant notes associated with fatty acids.

Higher alcohols form a large group of volatiles in wines, which at high levels contribute negatively to their aroma, except for 2-phenylethanol that produces “flowery” and “sweet” notes [30]. Different *Hanseniaspora* species may enhance the levels of higher alcohols in wines and have been shown to produce higher levels of 3-methyl-1-butanol and 2-methyl-1-butanol compared to *S. cerevisiae* [31]. *H. opuntiae* in particular was previously shown to increase 3-methyl-1-butanol and 2-phenylethanol [14]. On the contrary, in the present study, *H. opuntiae* decreased the levels of the latter alcohols, causing a drop in the total net content of higher alcohols, while it increased the levels of 1-propanol, 1-butanol, 2-methyl-1-propanol and the herbaceous C6 alcohols 1-hexanol, among others.

The formation of esters by yeasts plays a key role in the aroma profile of wines. Therefore, the ability of *Hanseniaspora* yeasts to produce high levels of esters is of particular interest in winemaking. *Hanseniaspora* species have been shown to increase the fruity esters in wines [31]. *H. uvarum*, for instance, increased acetate ester levels when used in fermentations, especially those of ethyl acetate, isoamyl acetate and 2-phenylethyl acetate [32]. *H. osmophila* and *H. vineae* were also associated with elevated acetate esters in wine, especially 2-phenylethyl acetate [33,34]. However, little is known about ester production of must fermented with *H. opuntiae*. Recently, del Fresno et al. [31] determined six esters in wines produced with a *H. opuntiae* strain as a co-inoculum and found that it produced higher levels of isobutyl acetate, isoamyl acetate and up to 2.8 times higher 2-phenylethyl acetate compared to *S. cerevisiae* alone. Importantly, this strain produced relatively low levels of ethyl acetate, which at high concentration may spoil wine. On the contrary, other strains of *H. opuntiae* formed elevated amounts of ethyl acetate, which may be regarded a spoilage factor when present in high amounts, along with high levels of isoamyl acetate [13]. *H. opuntiae* in SQ wine increased all acetate esters compared to IS wine, except for hexyl acetate and 3-hexenyl acetate. Prominent differences were reported for ethyl acetate and 2-phenylethyl acetate as also previously observed for other *Hanseniaspora* species. Particularly, the concentration of 2-phenylethyl acetate, a compound responsible for imparting flowery, rosy, and honey-like fragrances with fruity undertones, exhibited a 2.6-fold increase in SQ as compared to IS. Important increments were also observed for other significant acetate esters of wine, i.e., propyl acetate and isobutyl acetate, which highly confer to the fruity character. Consistent with these observations, the level of fruity and flowery scent in the SQ wine showed a notable increase in comparison to the other wines. The elevated levels of ethyl acetate caused a slight ‘pungent’ note in the SQ as compared to other ferments. Yet, the SQ wine was the most preferred among the accessors. Still, ethyl acetate levels should be considered if *H. opuntiae* is to be used in wine fermentations.

Although *Hanseniaspora* species commonly increase the acetate ester content, certain *H. uvarum* strains have been also shown to raise fatty acid esters in wine including ethyl hexanoate, ethyl octanoate and ethyl decanoate [32,35]. It was shown previously that *H. opuntiae* produced low levels of ethyl octanoate, ethyl decanoate and ethyl dodecanoate but high levels of ethyl butyrate [13]. In the present study, the total ethyl esters content was lower in SQ compared to IS. This difference in the net content was mainly ascribed to the significant decrease in ethyl decanoate, ethyl 9-decenoate, and ethyl octanoate in SQ compared to IS. Despite the decline in total ethyl ester content, significant increases in several compounds were noted, including ethyl butyrate, ethyl heptanoate and ethyl 7-octenoate, which are associated with fruity notes.

Terpenes have a significant role in wine aroma. They predominantly originate from grapes of muscat varieties and confer a floral and fruit character to the wine. *Hanseniaspora* species may increase the terpenic content of the wine by de novo synthesis of monoterpenes. Specifically, b-citronellol and a-terpineol were increased by 2-fold in *Hanseniaspora*-fermented wines compared to *Saccharomyces*-fermented wines [31]. *H. uvarum* may produce significantly higher levels of citronellol compared to *S. cerevisiae* in wine fermentation [23]. *H. opuntiae* was also shown to produce citronellol [13,15]. Here, *H. opuntiae* produced a significant amount of citronellol, which confers citronella, rose and green notes to wines. This is of particular benefit for improving the terpene content and the aroma profile of ‘Sideritis’, a non-aromatic grape variety.

The use of *Hanseniaspora* yeasts in wine production has been associated with several positive sensory attributes. For instance, wines produced with the addition of *H. uvarum* or *H. opuntiae* were characterized by ‘hazelnut’, ‘coffee’, ‘caramel’ and ‘cherry’ notes [13]. *H. opuntiae* was also shown to confer increased floral and sweet notes in wine [14]. In the present study, the panel of accessors favoured wines produced with *H. opuntiae* over others, due to the presence of increased floral and tree-fruits aroma, body, and after-taste, and a more favourable overall acceptance of the wine. A pungent character was slightly perceived possibly due to the higher levels of acetic acid and ethyl acetate in the SQ wines. It seems that *H. opuntiae* has a strong impact on the sensory profile of the wine like other *Hanseniaspora* spp., which can be largely explained by their increased enzymatic activity compared to *S. cerevisiae* and also other non-*Saccharomyces* species [4,27]. The present findings indicate that the inclusion of *H. opuntiae* in wine fermentation yeast inocula, in combination with *S. cerevisiae*, may increase the aroma intensity and intensify the floral and fruity aromas of Sideritis wine and potentially other non-aromatic grapes/wines. Additional studies considering different *H. opuntiae* strains and its further application at industrial-scale wine production will provide greater insights for its potential in winemaking.

## Figures and Tables

**Table 1:** Chemical characteristics of wines produced by different inoculation protocols (mean ± SD). Significant differences are indicated by different letters (*p* < 0.05).

Chemical Parameter	Inoculation Protocol ^1^			
	CS	IS	SQ	SP
Total acidity (as tartaric acid g/L)	4.88 ± 0.05 ^a^	4.82 ± 0.08 ^a^	4.82 ± 0.24 ^a^	4.56 ± 0.03 ^a^
pH	3.13 ± 0.01 ^a^	3.14 ± 0.01 ^a^	3.15 ± 0.0 ^a^	3.18 ± 0.02 ^a^
Volatile acidity (as acetic acid g/L)	0.35 ± 0.02 ^b^	0.64 ± 0.06 ^a^	0.77 ± 0.05 ^a^	0.63 ± 0.11 ^a^
Free SO_2_ (mg/L)	19.52 ± 4.07 ^a^	13.44 ± 1.81 ^ab^	3.84 ± 0.00 ^c^	7.04 ± 0.91 ^bc^
Total SO_2_ (mg/L)	90.24 ± 9.05 ^a^	90.88 ± 6.34 ^a^	112.00 ± 0.91 ^a^	106.43 ± 13.30 ^a^
Glucose (g/L)	0.06 ± 0.00 ^a^	0.06 ± 0.02 ^a^	0.04 ± 0.00 ^a^	0.06 ± 0.03 ^a^
Fructose (g/L)	0.13 ± 0.01 ^a^	1.04 ± 1.04 ^a^	0.72 ± 0.19 ^a^	1.47 ± 0.85 ^a^
Density	0.9902 ± 0.0002 ^a^	0.9900 ± 0.0004 ^a^	0.9902 ± 0.0000 ^a^	0.9901 ± 0.0000 ^a^
Lactic acid (g/L)	0.02 ± 0.00 ^a^	0.02 ± 0.00 ^a^	0.03 ± 0.00 ^a^	0.03 ± 0.00 ^a^
Malic acid (g/L)	0.37 ± 0.01 ^ab^	0.40 ± 0.0 ^a^	0.33 ± 0.00 ^b^	0.35 ± 0.02 ^ab^
Glycerol (g/L)	6.43 ± 0.18 ^a^	5.25 ± 0.07 ^b^	5.43 ± 0.04 ^b^	5.20 ± 0.00 ^b^
Citric acid (g/L)	0.14 ± 0.01 ^a^	0.13 ± 0.01 ^a^	0.12 ± 0.00 ^a^	0.12 ± 0.00 ^a^
Acetaldehyde (mg/L)	32 ± 2 ^c^	53 ± 4 ^bc^	100 ± 4 ^a^	85 ± 21 ^ab^
Ethanol (%vol)	10.9 ± 0.0 ^a^	10.9 ± 0.1 ^a^	10.8 ± 0.0 ^a^	10.9 ± 0.1 ^a^

^1^ CS: *S. cerevisiae* VIN13; IS: S. cerevisiae W7; SQ: *H. οpuntiae* L1 and *S. cerevisiae* W7 added sequentially; and SP: un-inoculated fermentation.

**Table 2:** Relative content (mg/L relative to internal standard) of volatile compounds in wines produced by different inoculation protocols (mean ± SD). Significant differences are indicated by different letters (*p* < 0.05).

Chemical Parameter	RID ^1^	RIref ^2^	RI ^3^	Inoculation Protocol ^4^			
				CS	IS	SQ	SP
**Acetate esters**							
Methyl acetate	A	828	820	0.45 ± 0.04 ^ab^	0.17 ± 0.03 ^b^	1.43 ± 0.02 ^a^	0.78 ± 0.59 ^ab^
Ethyl Acetate	A	888	878	196.56 ± 0.06 ^ab^	170.04 ± 3.31 ^b^	585.87 ± 8.70 ^a^	325.22 ± 200.21 ^ab^
Propyl acetate	A	973	956	1.65 ± 0.21 ^a^	0.37 ± 0.04 ^b^	1.18 ± 0.31 ^ab^	0.69 ± 0.32 ^ab^
Ιsobutyl acetate	A	1012	1001	2.64 ± 0.08 ^a^	1.05 ± 0.12 ^b^	2.41 ± 0.39 ^a^	1.64 ± 0.45 ^ab^
Ιsoamyl acetate	A	1122	1114	390.12 ± 19.83 ^a^	258.86 ± 41.75 ^ab^	265.27 ± 72.66 ^ab^	195.13 ± 10.83 ^b^
Hexyl acetate	A	1272	1267	145.47 ± 3.14 ^a^	104.41 ± 1.50 ^ab^	83.50 ± 12.23 ^b^	79.22 ± 12.38 ^b^
(*Z*)-3-Hexen-1-yl acetate	B	1306	1310	7.72 ± 0.10 ^a^	5.30 ± 0.10 ^b^	5.07 ± 0.76 ^b^	4.35 ± 0.12 ^b^
Heptyl acetate	B	1377	1368	0.46 ± 0.25 ^c^	2.24 ± 0.04 ^bc^	8.24 ± 1.43 ^a^	4.74 ± 1.04 ^ab^
2-Phenylethyl acetate	A	1813	1798	20.09 ± 0.26 ^a^	29.71 ± 0.69 ^a^	78.29 ± 10.04 ^a^	39.95 ± 28.37 ^a^
*Total*				*765.16*	*572.16*	*1031.27*	*651.71*
**Esters of fatty acids**							
Ethyl propionate	A	953	940	2.62 ± 0.07 ^a^	1.60 ± 0.11 ^a^	3.38 ± 0.59 ^a^	2.30 ± 0.70 ^a^
Ethyl isobutyrate	A	961	948	0.83 ± 0.02 ^a^	0.61 ± 0.10 ^a^	0.99 ± 0.18 ^a^	0.64 ± 0.26 ^a^
Ethyl butyrate	A	1035	1024	19.24 ± 0.10 ^b^	19.15 ± 1.16 ^b^	22.84 ± 0.61 ^a^	19.73 ± 0.62 ^b^
Ethyl isovalerate	C	1068	1057	0.95 ± 0.04 ^a^	0.55 ± 0.27 ^a^	0.30 ± 0.35 ^a^	0.41 ± 0.05 ^a^
Methyl hexanoate	C	1184	1180	0.54 ± 0.05 ^a^	0.71 ± 0.04 ^a^	0.57 ± 0.15 ^a^	0.56 ± 0.06 ^a^
Ethyl hexanoate	A	1233	1229	446.89 ± 28.80 ^a^	597.44 ± 37.57 ^a^	584.44 ± 32.13 ^a^	497.85 ± 62.39 ^a^
Ethyl 3-hexenoate	C	1290	1295	0.14 ± 0.04 ^a^	0.19 ± 0.03 ^a^	0.53 ± 0.35 ^a^	0.22 ± 0.05 ^a^
Ethyl heptanoate	A	1331	1328	ND ^5^	2.14 ± 0.01 ^b^	15.05 ± 2.17 ^a^	7.61 ± 3.82 ^ab^
Ethyl 2-hexenoate	B	1340	1338	2.48 ± 0.02 ^c^	5.91 ± 0.47 ^a^	4.70 ± 0.12 ^b^	3.24 ± 0.26 ^c^
Methyl octanoate	B	1385	1383	0.99 ± 0.01 ^b^	3.71 ± 0.03 ^a^	2.19 ± 0.45 ^ab^	2.02 ± 0.72 ^ab^
Ethyl octanoate	A	1435	1430	594.17 ± 27.70 ^c^	1679.17 ± 57.85 ^a^	1237.64 ± 129.32 ^ab^	1014.70 ± 187.98 ^bc^
Ethyl 7-octenoate	B	1478	1478	0.31 ± 0.01 ^c^	2.91 ± 0.31 ^b^	7.92 ± 0.17 ^a^	4.14 ± 0.87 ^b^
Ethyl nonanoate	A	1531	1529	0.21 ± 0.30 ^b^	1.50 ± 0.11 ^a^	1.78 ± 0.07 ^a^	1.79 ± 0.37 ^a^
Methyl decanoate	C	1593	1587	0.20 ± 0.11 ^b^	1.99 ± 0.10 ^a^	0.55 ± 0.41 ^b^	0.74 ± 0.35 ^b^
Ethyl decanoate	A	1638	1630	240.87 ± 44.62 ^b^	962.36 ± 14.41 ^a^	509.39 ± 5.74 ^b^	420.94 ± 134.91 ^b^
3-Methylbutyl octanoate	B	1658	1650	1.26 ± 0.40 ^b^	10.67 ± 0.01 ^a^	4.37 ± 0.19 ^b^	3.76 ± 2.23 ^b^
Diethyl succinate	A	1680	1665	0.34 ± 0.34 ^a^	0.68 ± 0.10 ^a^	0.54 ± 0.42 ^a^	0.65 ± 0.09 ^a^
Ethyl 9-decenoate	C	1694	1681	419.94 ± 14.34 ^b^	1046.28 ± 33.44 ^a^	548.04 ± 27.85 ^ab^	554.70 ± 259.64 ^ab^
3-Methylbutyl pentadecanoate	C	1863	1849	0.28 ± 0.17 ^b^	2.17 ± 0.13 ^a^	0.75 ± 0.16 ^b^	0.64 ± 0.46 ^b^
*Total*				*1732.29*	*4339.75*	*2945.98*	*2536.63*
**Alcohols**							
1-Propanol	A	1036	1032	4.95 ± 0.42 ^a^	2.72 ± 0.40 ^a^	4.08 ± 1.15 ^a^	3.49 ± 0.71 ^a^
2-Methyl-1-propanol	A	1092	1090	15.10 ± 0.30 ^b^	14.76 ± 0.07 ^b^	23.22 ± 2.47 ^a^	18.98 ± 0.12 ^ab^
1-Butanol	A	1142	1143	0.53 ± 0.00 ^a^	0.79 ± 0.11 ^a^	0.92 ± 0.19 ^a^	0.53 ± 0.23 ^a^
3-Methyl-1-butanol	A	1209	1207	530.22 ± 59.07 ^a^	474.83 ± 40.11 ^a^	374.15 ± 35.24 ^a^	438.70 ± 84.03 ^a^
2-Heptanol	C	1320	1319	ND	0.06 ± 0.00 ^b^	0.51 ± 0.02 ^a^	0.46 ± 0.10 ^a^
1-Hexanol	A	1355	1351	48.28 ± 0.21 ^b^	49.90 ± 0.59 ^b^	66.84 ± 0.69 ^a^	63.12 ± 1.78 ^a^
(*Z*)-3-Hexen-1-ol	B	1382	1380	4.15 ± 0.60 ^a^	3.54 ± 0.06 ^a^	3.26 ± 0.95 ^a^	3.60 ± 0.07 ^a^
1-Heptanol	B	1453	1452	4.68 ± 0.66 ^c^	15.43 ± 0.99 ^b^	30.45 ± 4.03 ^a^	28.92 ± 0.25 ^a^
2-Ethyl-1-hexanol	A	1491	1485	1.19 ± 0.06 ^a^	1.24 ± 0.16 ^a^	1.30 ± 0.06 ^a^	1.44 ± 0.02 ^a^
2-Nonanol	B	1521	1516	2.47 ± 0.61 ^a^	1.27 ± 0.03 ^b^	2.81 ± 0.14 ^a^	2.36 ± 0.15 ^a^
1-Octanol	A	1557	1553	6.25 ± 0.61 ^a^	3.14 ± 0.95 ^b^	2.45 ± 0.25 ^b^	2.74 ± 0.85 ^b^
2-Undecanol	C	1717	1711	0.44 ± 0.12 ^b^	ND	1.03 ± 0.08 ^a^	0.49 ± 0.14 ^b^
1-Decanol	B	1760	1753	2.20 ± 0.16 ^b^	4.95 ± 0.35 ^a^	3.23 ± 0.29 ^ab^	3.25 ± 0.84 ^ab^
2-Phenylethanol	A	1906	1894	52.54 ± 9.01 ^a^	111.53 ± 5.22 ^a^	66.78 ± 4.23 ^a^	68.83 ± 27.61 ^a^
1-Dodecanol	B	1966	1958	7.37 ± 0.61 ^ab^	6.07 ± 0.50 ^ab^	4.63 ± 0.04 ^b^	9.76 ± 1.70 ^a^
1-Tetradecanol	C	2165	2181	0.85 ± 0.14 ^a^	0.72 ± 0.74 ^a^	0.93 ± 0.05 ^a^	1.06 ± 0.50 ^a^
*Total*				*681.21*	*690.95*	*586.61*	*647.71*
**Carboxylic acids**							
Acetic acid	A	1449	1442	4.85 ± 0.03 ^b^	10.35 ± 0.33 ^a^	12.08 ± 0.84 ^a^	7.27 ± 0.97 ^b^
3-Methylbutanoic acid	C	1666	1660	1.12 ± 0.11 ^a^	1.32 ± 0.04 ^a^	0.67 ± 0.01 ^b^	0.74 ± 0.10 ^b^
Hexanoic acid	A	1846	1830	18.33 ± 1.14 ^b^	45.62 ± 2.80 ^a^	33.02 ± 7.71 ^ab^	31.27 ± 4.32 ^ab^
Octanoic acid	A	2060	2060	111.71 ± 9.49 ^b^	212.86 ± 14.60 ^a^	124.66 ± 29.27 ^ab^	140.02 ± 33.73 ^ab^
Nonanoic acid	B	2171	2174	7.39 ± 0.78 ^a^	13.96 ± 15.43 ^a^	4.92 ± 2.33 ^a^	2.98 ± 0.27 ^a^
Decanoic acid	B	2276	2269	53.40 ± 3.33 ^a^	94.87 ± 3.50 ^a^	44.54 ± 9.56 ^a^	60.24 ± 23.56 ^a^
9-Decenoic acid	B	2341	2333	15.22 ± 2.94 ^a^	18.80 ± 0.24 ^a^	8.64 ± 1.55 ^a^	11.60 ± 5.67 ^a^
*Total*				*212.01*	*397.76*	*228.52*	*254.12*
**Terpenoids**							
Linalool	B	1547	1542	0.66 ± 0.41 ^a^	1.04 ± 0.07 ^a^	0.75 ± 0.42 ^a^	0.91 ± 0.14 ^a^
Citronellol	C	1765	1755	0.32 ± 0.04 ^a^	ND	1.03 ± 0.04 ^a^	0.79 ± 0.69 ^a^
Geranyl acetone	C	1859	1840	2.00 ± 0.90 ^a^	1.83 ± 0.39 ^a^	1.61 ± 0.45 ^a^	1.50 ± 0.38 ^a^
*Total*				*4.16*	*4.32*	*4.50*	*4.42*
**Carbonyl compounds**							
Acetaldehyde	A	702	690	0.61 ± 0.34 ^a^	1.30 ± 0.28 ^a^	7.40 ± 2.44 ^a^	4.23 ± 3.50 ^a^
2-Heptanone	B	1182	1174	0.48 ± 0.01 ^a^	0.57 ± 0.04 ^a^	0.82 ± 0.13 ^a^	0.96 ± 0.20 ^a^
Octanal	B	1289	1281	0.28 ± 0.10 ^a^	0.32 ± 0.13 ^a^	0.50 ± 0.17 ^a^	0.32 ± 0.04 ^a^
Nonanal	B	1391	1385	8.48 ± 4.22 ^a^	13.51 ± 10.79 ^a^	10.40 ± 2.93 ^a^	6.58 ± 1.89 ^a^
Decanal	B	1498	1490	0.65 ± 0.29 ^b^	1.67 ± 0.40 ^ab^	2.05 ± 0.03 ^a^	1.64 ± 0.20 ^ab^
Dodecanal	C	1711	1698	0.10 ± 0.05 ^a^	0.67 ± 0.16 ^a^	0.56 ± 0.17 ^a^	0.19 ± 0.16 ^a^
*Total*				*10.61*	*18.04*	*21.74*	*13.91*
**Other compounds**							
2-Methoxy-4-vinylphenol	C	2188	2194	ND	0.87 ± 0.13 ^a^	0.97 ± 0.16 ^a^	0.77 ± 0.02 ^a^

^1^ RID: reliability of identification. ^2^ Riref: retention index from Nist14 for DB-Wax column. ^3^ RI: retention index.^4^ CS: *S. cerevisiae* VIN13; IS: *S. cerevisiae* W7; SQ: *H. οpuntiae* L1 and *S. cerevisiae* W7 added sequentially; SP: un-inoculated fermentation.^5^ ND: not detected (signal to noise < 3).

## Data Availability

The original contributions presented in the study are included in the article, further inquiries can be directed to the corresponding author.
